# An analysis of mutational signatures of synonymous mutations across 15 cancer types

**DOI:** 10.1186/s12881-019-0926-4

**Published:** 2019-12-09

**Authors:** Yannan Bin, Xiaojuan Wang, Le Zhao, Pengbo Wen, Junfeng Xia

**Affiliations:** Institutes of Physical Science and Information Technology, School of Computer Science and Technology, Anhui University, Hefei, 230601 Anhui China

**Keywords:** Cancer, Synonymous mutations, Hotspot, Driver

## Abstract

**Background:**

Synonymous mutations have been identified to play important roles in cancer development, although they do not modify the protein sequences. However, relatively little research has specifically delineated the functionality of synonymous mutations in cancer.

**Results:**

We investigated the nucleotide-based and amino acid-based features of synonymous mutations across 15 cancer types from The Cancer Genome Atlas (TCGA), and revealed novel driver candidates by identifying hotspot mutations. Firstly, synonymous mutations were analyzed between TCGA and 1000 Genomes Project at nucleotide and amino acid levels. We found that C:G → T:A transitions were the most frequent single-base substitutions, and leucine underwent the largest number of synonymous mutations in TCGA due to prevalent C → T transition, which induced the transformation between optimal and non-optimal codons. Next, 97 synonymous hotspot mutations in 86 genes were nominated as candidate drivers with potential cancer risk by considering the mutational rates across different sequence contexts. We observed that non-CpG-island GC transition sequence context was positively selected across most of cancer types, and different sequence contexts under which hotspot mutations occur could be significance for genetic differences and functional features. We also found that the hotspots were more conserved than neutral mutations of hotspot-mutation-containing-genes and frequently happened at leucine. In addition, we mapped hotspots, neutral and non-hotspot mutations of hotspot-mutation-containing-genes to their respective protein domains and found ion transport domain was the most frequent one, which could mediate the cell interaction and had relevant implication for tumor therapy. And the signatures of synonymous hotspots were qualitatively similar with those of harmful missense variants.

**Conclusions:**

We illustrated the preferences of cancer associated synonymous mutations, especially hotspots, and laid the groundwork for understanding the synonymous mutations act as drivers in cancer.

## Background

Synonymous mutations, which occur in the gene-coding regions without changing the encoded amino acids, have long been supposed to be silent for the fitness of organisms and neutral during evolution [1]. However, this conservative concept begins to be rebutted by two lines of evidence: first one is the understanding of synonymous mutational effect on protein synthesis and folding; second, codon usage bias reveals that synonymous codons are under evolutionary pressure [2]. Because of the degeneracy of the genetic codons, synonymous mutations don’t changing the encoded amino acids, but change the DNA and RNA sequence. Nevertheless, there are growing evidences that the significant impact of synonymous mutations on RNA splicing, stability and folding [3, 4], translation or co-translational protein folding [5–8]. Chen et al. conducted a broad survey of 21,429 disease-related single nucleotide polymorphisms (SNPs) to indicate that synonymous SNPs and non-synonymous SNPs showed similar probability and effect size for human diseases [9]. In addition, some studies have identified that synonymous mutations frequently act as driver mutations in human cancers [10, 11] and can affect clinical outcome or treatment response [12–14].

As a complex genetic disease, cancer was affected by a large number of variants. But to date, the targets of drugs and treatments associated with cancer are limited on a few genes, therefore, it is difficult to achieve cures for cancer. Next-generation sequencing technology has enabled the systemic analyses of huge variants in large cohorts of cancer cases, e.g., The Cancer Genome Atlas (TCGA) [15] and International Cancer Genome Consortium [16]. Cancer genomes not only contain cancer-causing driver mutations, but also many additional accumulated passenger mutations without direct relation to the tumor phenotype. It is a key step to identify driver mutations for understanding cancer biology and evolving targeted treatments. There were several methods focused on predicting driver mutations, such as E-Driver [17], MuSiC [18] and OncodriveCLUST [19]. Nevertheless, those studies mainly focused on the missense mutations and ignored the potential functions of synonymous mutations. Although Silent Variant Analyzer [20] is a tool for the annotation and prediction of pathogenic synonymous mutations, the small datasets for training and validation restrict its applicability. TCGA, using the latest sequencing and analysis methods to identify somatic variants across thousands of tumors, is found to meet the data needs in this work [15, 21]. Additionally, more recent studies have indicated that different substitution types, codon usage bias and hotspot mutational positions in base sequence could be associated with different biological processes and cancer types [2, 22, 23]. The hotspot mentioned in this work is not the hotspot in protein-protein interfaces [24], and is defined as the mutation that occurs significantly more frequently than the background frequency characterized by genes, cancer types and mutation subtypes.

In this study, we documented the full repertoire of cancer associated synonymous mutations, especially synonymous hotspot mutations, to investigate the mutational signatures in cancer. To acquire insight into the characters of pathogenic and neutral synonymous mutations between cancer and benign samples, the differences of synonymous mutations at nucleotide and amino acid levels (such as nucleotide substitutions, mutational positions of codon and distribution of amino acids at which synonymous mutations happened) were investigated between datasets in TCGA and the 1000 Genomes Project (1000G) (as neutral samples) [16]. And then, we nominated synonymous hotspot mutations as candidate drivers based on the mutational rates across different sequence contexts and investigated the features (such as conservation, distribution of amino acids and protein domains undergo mutations) of hotspots, neutral synonymous mutations and non-hotspots in the hotspot-mutation-containing-genes (HMCGs). For the comprehensiveness of analysis, this study not only highlights the nucleotide level preferences, but also amino acids level, and especially hotspot mutations. The observation could add perspective to understand cancer-related synonymous mutations. The procedure is illustrated in Additional file 1: Figure S1.

## Material and methods

### Synonymous mutation dataset

The cancer related synonymous mutations in TCGA were downloaded from COSMIC v79 (Catalogue of Somatic Mutations in Cancer) [25]. We got 373,434 cancer related synonymous mutations obtained from 5749 tumor samples across 15 types of cancer: breast cancer (BRCA), central nervous system tumor (CNST), cervical adenocarcinoma (CEAD), endometrial adenocarcinoma (ENAD), haematopoietic and lymphoid tumor (HLTU), kidney carcinoma (KICA), large intestine adenocarcinoma (INAD), liver carcinoma (LICA), lung adenocarcinoma (LUAD), ovarian carcinoma (OVCA), prostate adenocarcinoma (PRAD), skin cancer (SKCA), stomach adenocarcinoma (STAD), thyroid carcinoma (THCA) and urinary tract carcinoma (UTCA).

The aim of 1000G is to discover variants with a frequency of occurrence > 1% in multiple human populations worldwide. In this study, 21,121 putatively benign synonymous mutations were derived from 1000G (the phase 3 version 5b, 20,130,502) and this dataset will be referred to as the neutral synonymous mutations dataset for the comparative analysis of cancer related synonymous mutations.

### Statistical analyses

The majority of statistical analyses in this work were completed by using R (https://www.r-project.org/), e.g., the distributions of synonymous variants across different cancer types, nucleotide substitutions and amino acids. Other statistical analyses were performed by GraphPad Prism 5 (GraphPad Software). A *p*-value < 0.05 was considered statistically significant.

### Hotspot mutations identification

Here we used the Hot-Driver package [26] to identify the hotspot mutations that are positively correlated with the number of mutations across all cancer samples for all 15 cancer types. The Hot-Driver suite assigns mutations to six subtypes: AT transition (ATts), AT transversion (ATtv), non-CpG-island GC transition (NC_GCts), non-CpG-island GC transversion (NC_GCtv), CpG-island GC transition (C_GCts) and CpG-island GC transversion (C_GCtv). Based on mutational significance of each mutation subtype on amino acid position, a statistical method combines the significance level of different mutation subtypes to calculate the overall *p*-values (Poisson distribution and Fisher’s test). We reported hotspot mutations in amino acid position with adjusted *p*-values < 0.05 corrected by false discovery rate. Lastly, in this study, to avoid the bias of background number of passenger mutations, we only selected the hotspot mutations that predicted as the pathogenic mutations by Functional Analysis through Hidden Markov Models [25].

To further investigate the mutational signatures of hotspot mutations, neutral synonymous mutations of HMCGs in 1000G and the non-hotspot mutation of HMCGs in TCGA were used as control datasets. These three datasets were applied for the further functional analyses, for example, conservation, amino acids and protein domains under which mutations occurred.

### Conservation comparison

The conservation of nucleotide sequence for each gene was assessed by rejected substitution (RS) score, computed by GERP++ [27]. In this work, RS scores were extracted from the nucleotide bases that belong to hotspot mutations, neutral synonymous mutations of HMCGs in 1000G and non-hotspot mutations of HMCGs in TCGA, respectively. Single-tailed unpaired t-test was used to test significantly difference between hotspot mutations, neutral synonymous mutations and non-hotspot mutations.

### Protein domain annotation

We mapped the hotspot mutations to conserved protein domains obtained from Pfam-A (version 29.0), a database of protein domain families [28], and manually curated data were used in this work. Since genes that shared a common domain are more likely to share related functions, the important mutations in certain genes tend to cluster in close proximity within functional domains [18, 29, 30].

## Results and discussion

### Synonymous mutation distribution across cancer types

**Fig. 1:**
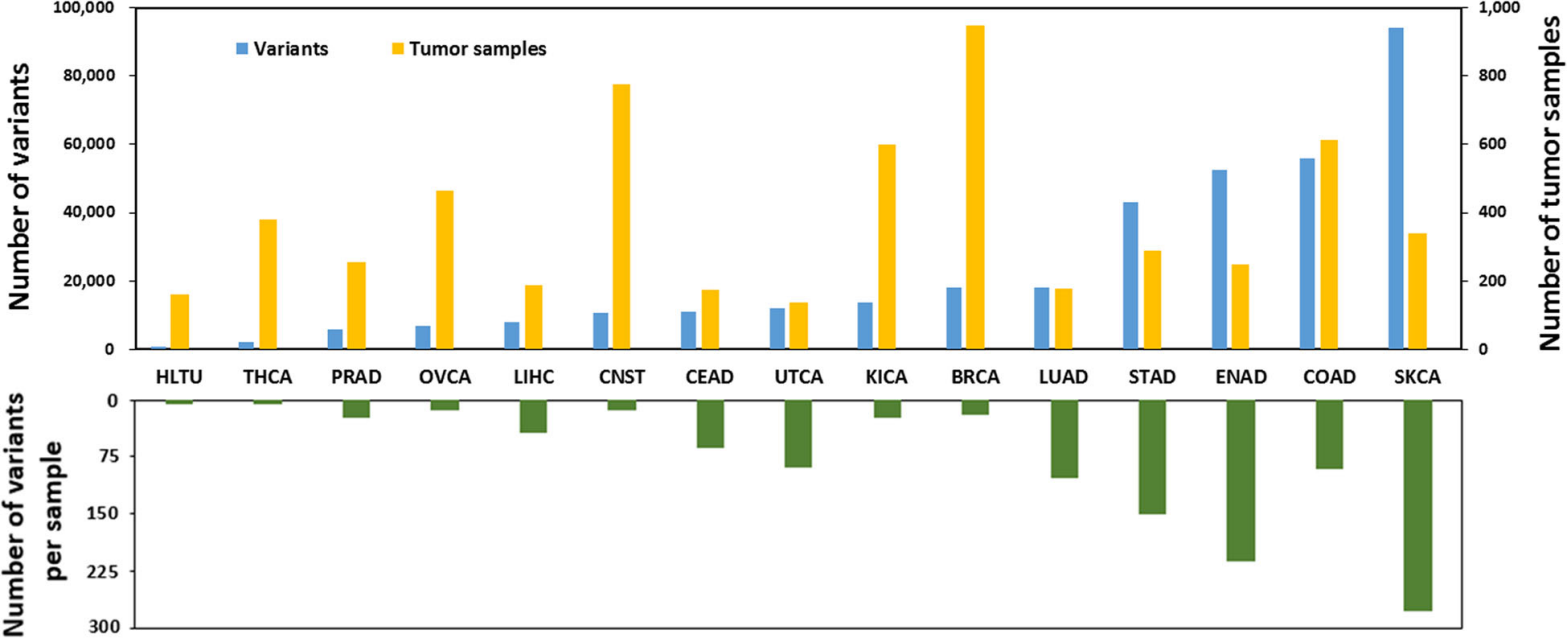
Distribution of synonymous variants, tumor samples across different cancer types. Upper panel: blue and yellow bars represent the proportions of synonymous variants and corresponding tumor samples, respectively. Lower panel: Green bar represents the average numbers of variants per sample. The x-axis represents the cancer types

From TCGA, we obtained 373,434 synonymous mutations of 5749 tumor samples from 15 types of cancer. As seen in the upper panel of Fig. 1, with regard to synonymous mutation proportion (blue bar), SKCA is the largest and HLTU the smallest. For the proportion of tumor samples (yellow bar), BRCA is the most and UTCA the fewest. Moreover, the average numbers of variants per sample across 15 cancer types are different with each other (the lower panel of Fig. 1), and exhibit many more or many less synonymous mutations per sample than the average number of 15 cancer types. Notable among these outliers are SKCA, ENAD, STAD and LUAD, which contain more than 100 synonymous mutations per sample. These larger numbers of mutations reflect the participation of potential factors (ultraviolet light, hyperestrogenism, *Helicobacter pylori* infection and cigarette smoke, respectively) in the pathogenesis of these cancer types [22, 31–33]. Due to ultraviolet light and deamination processes, the majority of SKCA mutations are C:G → T:A transitions [22]. Additionally, it has been reported that the mutations occur at methylated CpG dinucleotide, majority of which are C:G → T:A transitions, would significantly cause human genetic diseases [34]. Studies have shown that nucleotide substitutions, including synonymous mutations, could be related to carcinogen exposures and DNA repair processes [35–37].

### Synonymous mutational nucleotide substitutions

**Fig. 2:**
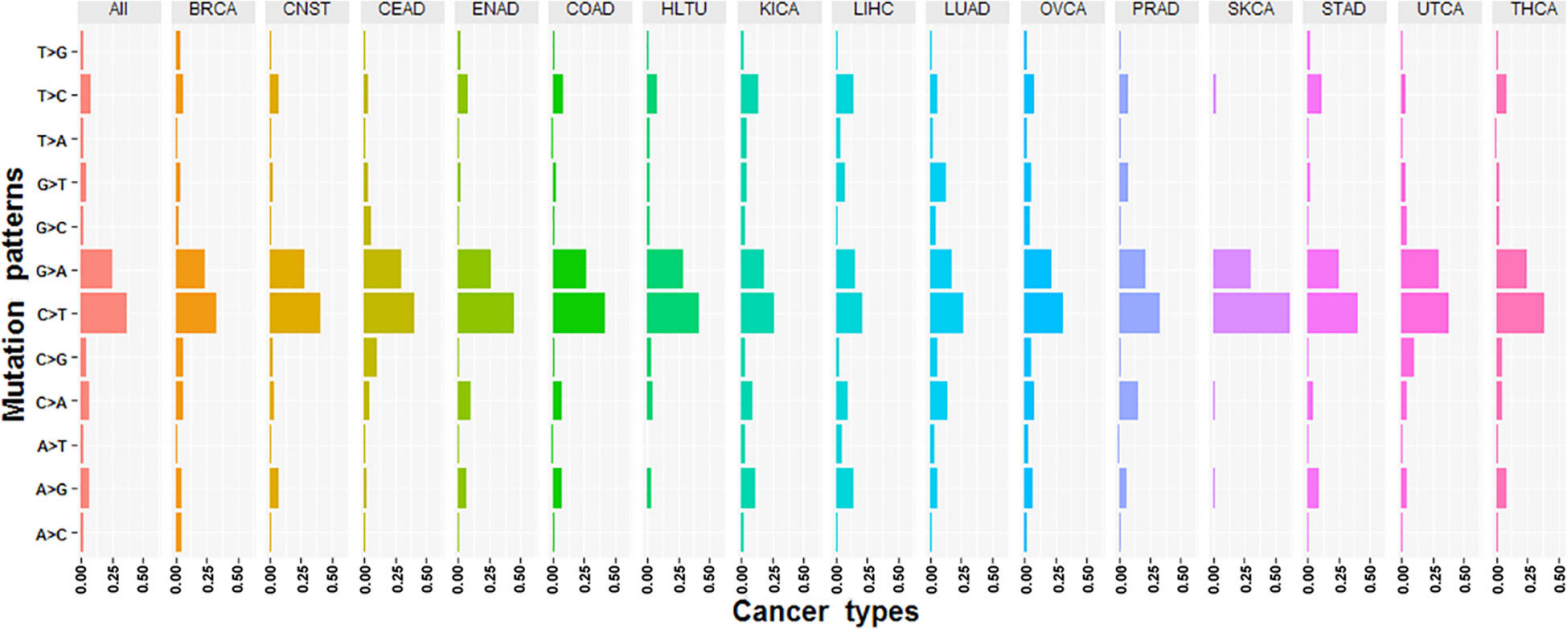
Distribution of 12 possible mutation patterns of synonymous mutations in different cancer types. The x-axis represents the cancer types and the y-axis represents the 12 mutation patterns. Each bar represents the percentage of mutational types. ‘All’ in this figure represents the average proportions of mutational types across 15 cancer types

Distribution of 12 possible mutational nucleotide substitution patterns of synonymous mutations across the 15 cancer types was shown in Fig. 2. The greatest frequently occurring is C → T transitional substitution (with average proportions of 44.01% across 15 cancer types), which is possible to associate with the aberrant DNA methylation [38–40]. SKCA contains the largest proportion of C → T transitions than other cancer types, owing to the signatures of ultraviolet light exposure and deamination processes [22]. Among transversions, the C → A substitution is the most frequent one (6.18%). As a result of signature smoke exposure, LUAD has an increased C → A transversions [33]. At 5-methylcytosine in CpG dinucleotides, C:G → T:A transitions and C → A transversion are associated with the most common epigenetic modifications of DNA [34, 35]. Moreover, due to the overabundance of synonymous sites involved in CpG dinucleotides, the mutation rate in exons is 30~60% higher than that in the non-coding regions [41]. It is found that the percentage of transitions between C and T preceded that between A and G, which is known to be a general property of DNA sequence change and evolution [42]. Moreover, the most frequently substituted bases are C and G, and the most frequently mutated to bases are T and A. Cancer associated synonymous mutations have the tendency to become A/T-rich. Previous study has proposed that special A/T-rich sequence binding protein acts as a global chromatin organizer for metastatic activity by controlling gene expression [43].

### Comparisons between TCGA and 1000G datasets

**Fig. 3:**
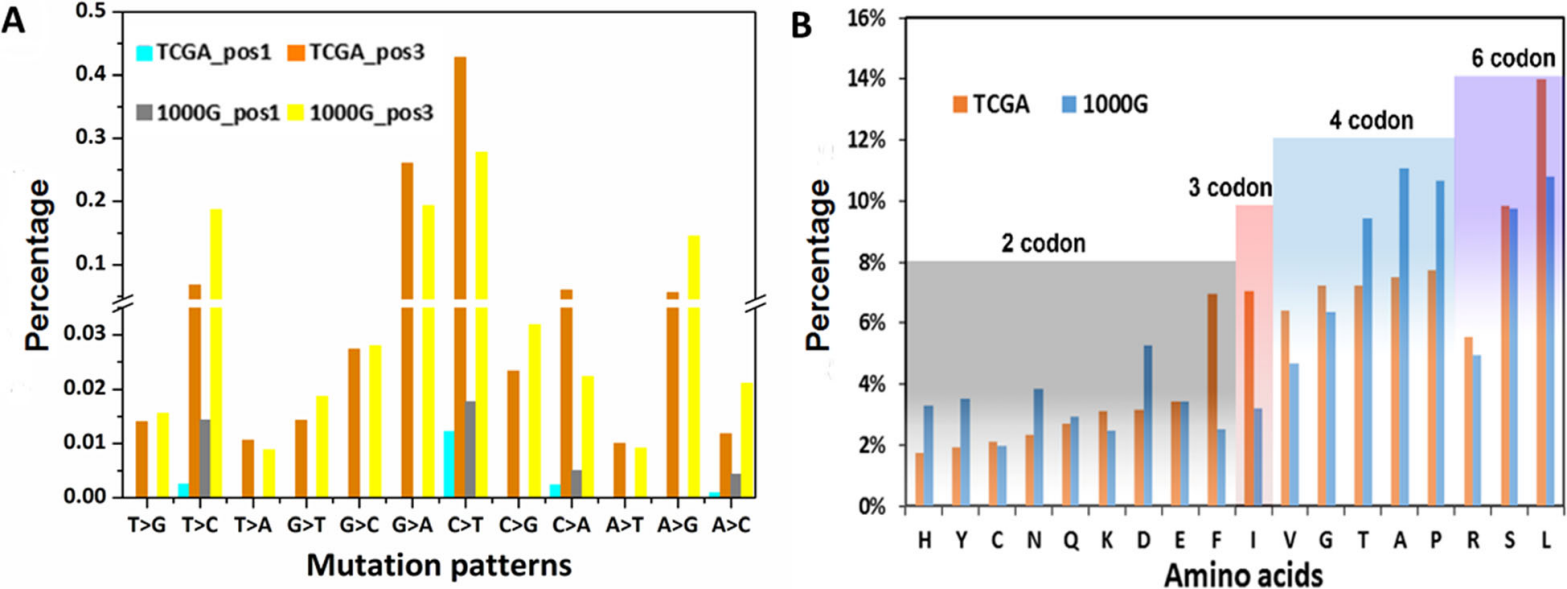
Mutational signatures of synonymous mutations in TCGA and 1000G. **a** Distribution of synonymous mutations for the 12 mutation patterns at pos1 and pos3 of codons in TCGA and 1000G. **b** Proportions of 18 amino acids with two, three, four or six-fold degenerate codons under which synonymous mutations occurred. Orange bar represents the proportion of mutations in TCGA and grey bar represents that in 1000G. 2, 3, 4, and 6 codon represent the numbers of synonymous codons, respectively

At nucleotide level, owing to the degeneracy of genetic codons, the nucleotide substitutions of synonymous codons occur at the third codon position (pos3), except some L and R codons (only T↔C transition and A↔C transversion) vary at the first codon position (pos1) (Additional file 1: Table S1). Similar with the distributions of mutational types in TCGA dataset, the most frequency mutational nucleotide changes in 1000G dataset are also C → T and G → A transitions (Fig. 3a). However, there are some differences for proportions of mutational nucleotide changes between TCGA and 1000G datasets. Firstly, we investigated the differences by performing a one-sample t-test, and the average proportion of each substitution in 1000G dataset was used as hypothetical value. A *p*-value < 0.05 is considered to be significant. The distributions of single-base mutational nucleotide changes in TCGA dataset are significant different from those in 1000G datasets (*p*-value < 0.001) except T → G transversion at pos3 (*p*-value = 0.8066). The non-significant different may be due to the less effect of T → G transversion on the transformation between efficient codon and lower efficient codon, which can affect protein production. Secondly, based on the significant differences, the single-base substitutions in TCGA dataset are apt to G:C → A:T transitions, in contrast, these substitutions in 1000G are T:A → C:G transitions. Synonymous mutations in TCGA are prone to become A/T-rich. In a previous study, it was reported that A/T-rich sequence could affect gene expression and be more important for cancer development [43].

Besides nucleotide level, the analysis of synonymous mutations was also performed at the amino acid level. Except Met (M) and Trp (W), all amino acids are encoded by two or more codons. The correlation between the percentages of mutation and the codon numbers of amino acids in TCGA (*r*^2^ = 0.67) is stronger than that in 1000G (*r*^2^ = 0.54) (Additional file 1: Figure S2). DNA sequences of diverse organisms have shown that synonymous codons of amino acids are used with unequal frequency [44]. The codon usage bias is related to various biological processes, such as gene expression level, protein structure, mutation frequency and GC composition [45]. However, it is probable that the uses of synonymous codons in TCGA tend to be at equal frequencies, and are less affected by codon usage bias than those in 1000G. Furthermore, the efficient codon replacing with a less efficient one could affect protein synthesis. Because the abundance of cognate tRNAs involved in preferred codons are available within the cell, the use of efficient codons could increase the gene expression [6]. Therefore, it is proposed that the cancer related synonymous mutations prefer to influence the gene expression and are more pathogenic than neutral ones in TCGA.

In TCGA and 1000G datasets, the mutational proportions of 18 amino acids with two, three, four or six-fold degenerate codons are different with each other (Fig. 3b). In 1000G, Ala is the most frequently mutated amino acid due to G → A transition. And this transition is associated with two of four Ala’s codons, but independent of the transformation between optimal and non-optimal codons [10]. In TCGA, synonymous mutations are dominated by Leu (L) due to prevalent C:G → T:A transitions. It is similar with the character of pathogenic missense mutations, the substitutions under L are also the most frequent [46]. It is notable that among the three amino acids with six synonymous codons, Arg (R) shows the fewest number of mutations not only in TCGA but also in 1000G, which may be associated with the synonymous codon usage bias (R has only one optimal codon while Ser and L both have two optimal codons). In summary, it is possible that synonymous mutations under L may have more important effect on gene expression and protein production than the mutations of other amino acids during biological processes.

### Synonymous hotspot mutations for cancer

In this work, the hotspot mutation is defined as the mutation that occurs significantly more frequently than the background frequency characterized by genes, cancer types and mutation subtypes. In this study, we identified 97 hotspot mutations in 86 HMCGs associated with 14 cancer types (Additional file 1: Table S2). There is none hotspot mutation in HLTU due to the lowest mutation frequency (Fig. 1). To investigate the differences across cancers, we compared the number of synonymous hotspot mutations in different cancer types. From the distribution of hotspot mutations across cancers (Additional file 1: Figure S3A), it was found that the number of hotspot mutations varies largely from one cancer to another, many more or many fewer mutations than average. For example, INAD has the largest number of hotspots (39 hotspots), but ENAD and STAD only have 13 hotspots with the smallest number, and HLTU has none. The enrichment of hotspot mutations reflects the genetic heterogeneity of INAD, which has been discussed in previous research [47]. And heterogeneity may affect the progression of INAD from the early to the advanced stages, drive phenotypic variations and present a significant challenge to personalized medicine. By contrast, HLTU has none hotspot and THCA has only one hotspot mutation in *ILF3* (N192). In addition, to estimate the important synonymous mutations for pan-cancer, the distribution of hotspot mutations across different genes was analyzed (Additional file 1: Figure S3B). T125 in *TP53* is the most prevalently occurred mutation in nine different cancer types. It has been identified to be pathogenic for its detrimental role in *TP53* splicing [48]. It is also found that most hotspots are unique for only one cancer type. The common and diverse mutational signatures of hotspots across different cancer types may promote the understanding of the positive selection in the human genome, and facilitate the cancer target therapy [26].

**Fig. 4:**
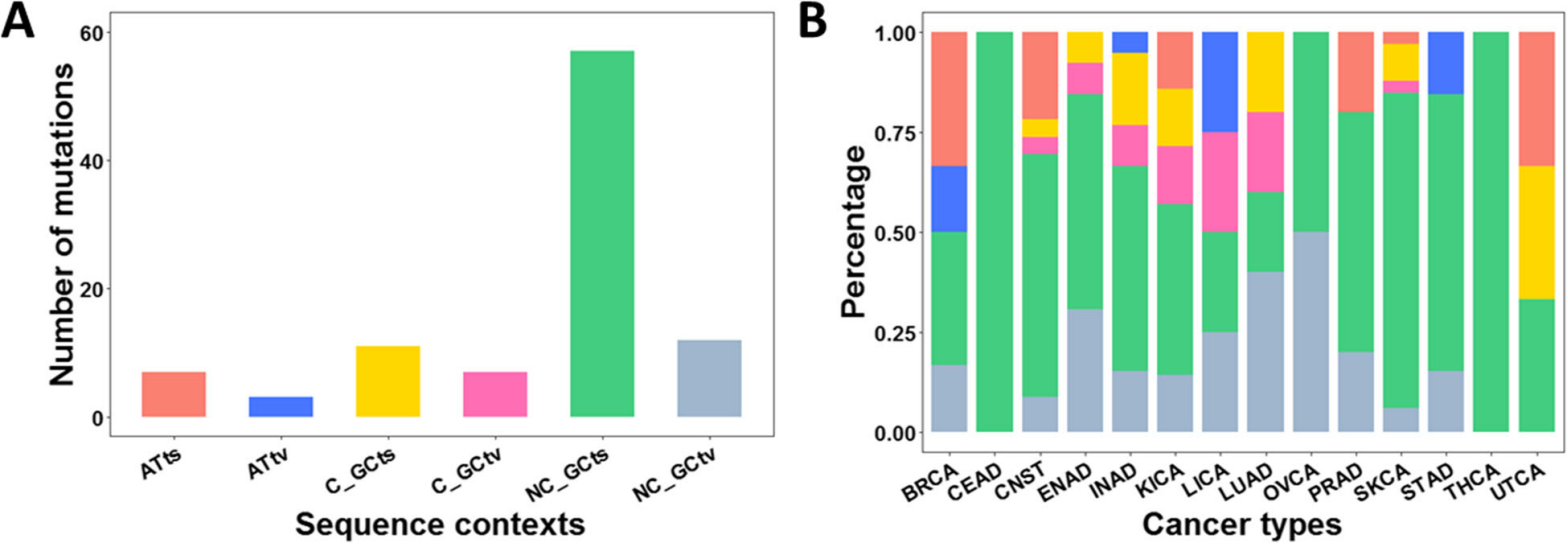
Mutational signatures of synonymous hotspot mutation in 15 cancer typers. **a** Histogram of the number of hotspot mutations under different sequence contexts. **b** Bar diagram where the value 100% represents the total number of mutations under different sequence contexts distributed in each cancer type. ATts, AT transition (coral bar in **a** and **b**); ATtv, AT transversion (blue bar in **a** and **b**); C_GCts, CpG-island GC transition (yellow bar in **a** and **b**); C_GCtv, CpG-island GC transversion (rose bar in **a** and **b**); NC_GCts, non-CpG-island GC transition (green bar in **a** and **b**); NC_GCtv, non-CpG-island GC transversion (grey bar in **a** and **b**)

The mutational characters of 97 hotspot mutations across 14 cancer types were also investigated under different sequence contexts, including ATts, ATtv, C_GCts, C_GCtv, NC_GCts and NC_GCtv six subtypes. From the distribution of hotspot mutations under different subtypes of sequence contexts (Fig. 4a), the number of hotspot mutations under NC_GCts sequence context (consists of C:G → T:A transitions) is the largest one compared with the other types, and that under ATtv (consists of A:T → C:G and A↔T transversions) is the least one. This phenomenon is due to the most frequency of C → T and G → A transitions and least frequency of A:T → C:G and A↔T transversions (Fig. 3a), which correspond to the NC_GCts and ATtv sequence contexts, respectively. As seen in Fig. 4b, the most widespread sequence context undergoes hotspots is NC_GCts sequence context, which presents in 14 cancer types. And it is also the most prevalent sequence context in nine cancer types (CEAD, CNST, ENAD, INAD, KICA, PRAD, SKCA, STAD and THCA). Moreover, in CEAD and THCA, the hotspots are only enriched in NC_GCts. However, in LUAD, the hotspot mutations are enriched in NC_GCtv sequence context. In LICA, OVCA and UTCA, the sequence contexts under which hotspot mutations occur are equal. In summary, NC_GCts sequence context is positively selected across most cancer types, and different sequence contexts on which hotspots happen are significant for considering their genetic differences and functional features.

### Conservation comparison

**Fig. 5:**
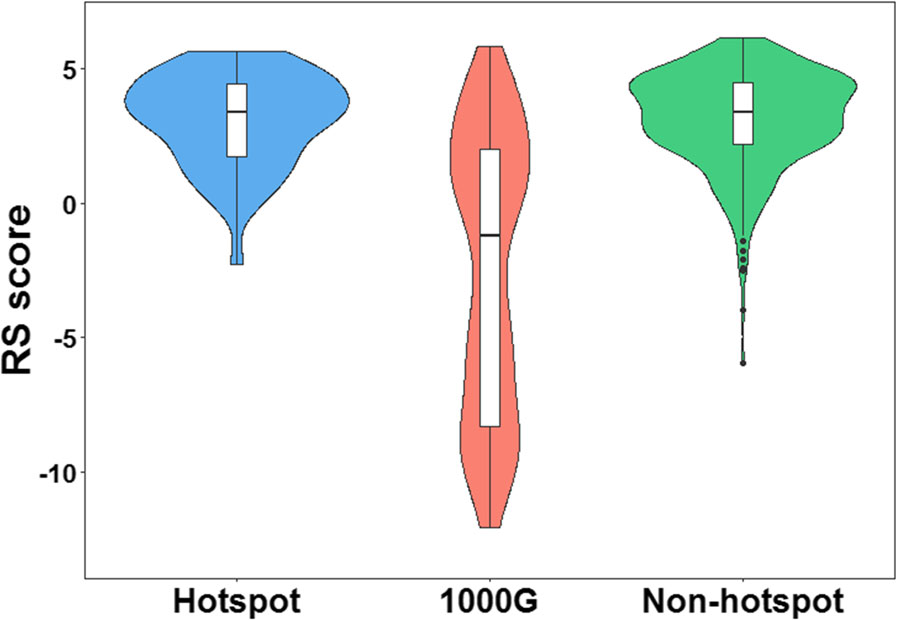
Comparison of RS scores between the datasets of hotspots and neutral synonymous mutations, non-hotspots. Hotspot represents hotspot mutations; 1000G represents neutral synonymous mutations of HMCGs in 1000G dataset; Non-hotspot represents non-hotspot mutations of HMCGs in TCGA dataset. The RS score of hotspot mutation dataset is significant higher than that neutral synonymous mutation dataset (*p*-value <2e-16). But there is no significant different between hotspot and non-hotspot datasets (*p*-value = 0.93) for RS score

It is customary for mutations with important functional and evolutionary implications located in highly conservative region and protein domains. To evaluate the conservation of hotspot mutations, neutral synonymous mutations of 86 HMCGs in 1000G (235 neutral synonymous mutations were shown in Additional file 2: Table S3) and non-hotspot mutations of HMCGs in TCGA (1358 non-hotspot mutations were shown in Additional file 3: Table S4), we computed their RS scores to estimate the evolutionary constraints across different genome sites. As shown in Fig. 5, the RS scores of hotspot mutations are significantly higher than those of neutral synonymous mutations in 1000G (*p*-value <2e-16). In contrast, there is no significantly different between hotspot and non-hotspot mutations (*p*-value = 0.93), it is possible that the non-hotspot in TCGA might influence cancer processes, but their harmfulness is less than that of hotspots. The result suggests that the sites which hotspots occur on are more conservative than those of neutral synonymous mutations. As potential driver mutations, these hotspots may be more important for cancer development.

### Amino acids analysis

**Fig. 6:**
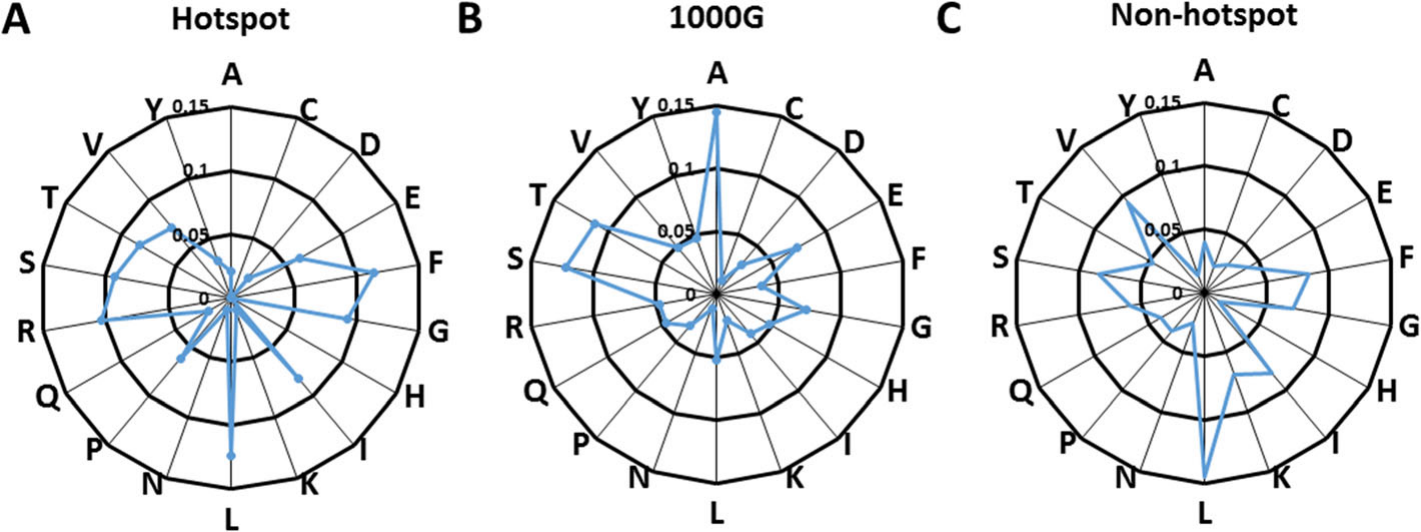
Radar diagrams showing the distributions of amino acid at which mutations occurred. **a** Hotspot mutations; **b** Neutral synonymous mutations of HMCGs in 1000G dataset; **c** Non-hotspot mutations of HMCGs in TCGA dataset

To further investigate the difference among hotspot mutations, neutral synonymous mutations and non-hotspot mutations of HMCGs, the distributions of amino acids under which the mutations occurred were investigated (Fig. 6). There is none synonymous mutation under Met and Trp due to the lack of synonymous codons. Clearly, the distributions of amino acids are different for the three synonymous mutation datasets. In hotspot mutation dataset, L and Phe (12.37 and 11.34%, respectively) are the most mutated amino acids due to prevalent C → T transition, which is involved the transformation between optimal and non-optimal codons. As an important amino acid of leucine-rich repeats, L is associated with a versatile structural framework for the formation of protein-protein interactions [49]. However, for neutral mutation dataset, the most frequent substitutions are under Ala (14.44%), which is largely tolerated outside functional site as the smallest residues can be fitted into structures easily. Among non-hotspot mutations, the most frequent substitutions are under L (14.51%). The distributions of the hotspot and neutral synonymous mutations are quantitatively similar to those of missense mutations published previously [46].

### Domain characterization

**Fig. 7:**
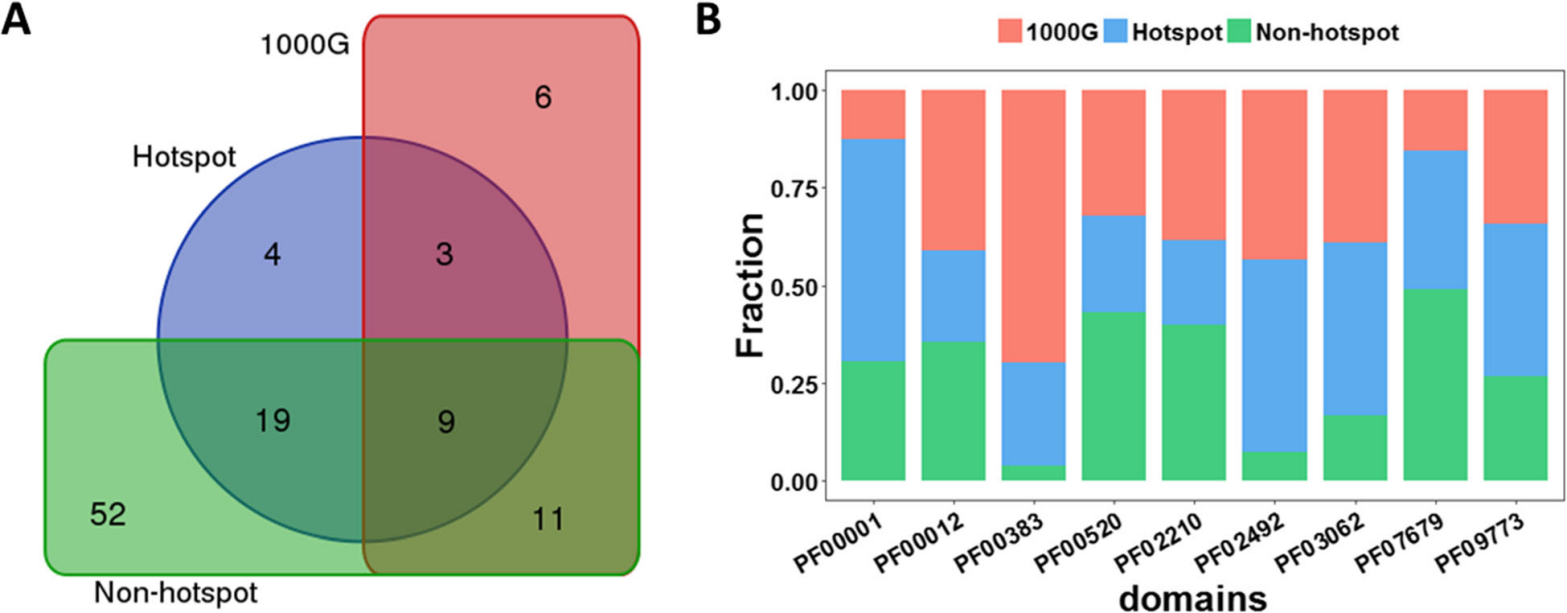
Analysis of mutation distribution in protein domains. **a** Venn diagram showing the number of domain types for the multiple intersections among hotspot mutations, neutral synonymous mutations and non-hotspot mutations. **b** Bar diagram where the value 100% represents the total number of mutations in the nine protein domains common to hotspot, neutral synonymous and non-hotspot mutations. Blue, salmon and green sections on the bar represent which proportions of the total mutations are hotspot, neutral synonymous and non-hotspot mutations, respectively. PF00001, 7tm_1 domain; PF00383, dCMP_cyt_deam_1 domain; PF02492, cobW domain; PF00012, HSP70 domain; PF00520, Ion_trans domain; PF07679, I-set domain; PF00055, Laminin_N domain; PF03062, MBOAT domain; PF09773, Meckelin domain

We also investigated the domain compositions of the proteins, under which hotspot mutations, neutral synonymous mutations of HMCGs in 1000G and non-hotspot mutations of HMCGs in TCGA occurred. Thirty-five different Pfam domains were detected in the proteins under which the hotspot mutations occurred, whereas 29 and 91 protein domains under which neutral synonymous and non-hotspot mutations happened, respectively. It is found that nine domain types are common to hotspot, neutral synonymous and non-hotspot mutations (Fig. 7a), including 7 transmembrane receptor domain (7tm_1, PF00001), cytidine and deoxycytidylate deaminase zinc-binding region domain (dCMP_cyt_deam_1 domain, PF00383), nucleotide-binding domain (cobW domain, PF02492), Hsp70 protein domain (HSP70 domain, PF00012), ion transport protein domain (Ion_trans domain, PF00520), immunoglobulin I-set domain (I-set domain, PF07679), laminin N-terminal domain (Laminin_N domain, PF00055), membrane-bound O-acyltransferase family domain (MBOAT domain, PF03062) and transmembrane protein 67 domain (Meckelin domain, PF09773). The functions of these domains are different from each other. As rhodopsin-like receptor, 7tm_1 domain comprises the group of G protein-coupled receptor and encompasses a wide range of functions such as various autocrine, paracrine, and endocrine processes. dCMP_cyt_deam_1 domain is the cytidine and deoxycytidylate deaminase zinc-binding region, which is associated with the catalytic activity of cytidine deaminase. cobW domain contains a nucleotide-binding loop and a histidine-rich region that plays an important role in metal binding. HSP70 domain is strongly upregulated by heat stress and toxic chemicals, particularly heavy metals. Ion_trans domain contains sodium, potassium and calcium ion channels, and a loop flanked by two helices determines ion selectivity. I-set domain is not only frequent in cell adhesion protein, but also appears in many other types of proteins [50]. Laminin_N domain is extracellular matrix molecule and MBOAT domain contains various acyltransferase enzymes. Then we analyzed the distributions of hotspot, neutral synonymous and non-hotspot mutations under the nine common protein domains. Among these nine domains, the Ion_trans domain is the most frequent one (28 items), but the proportion of hotspots is less than those of neutral synonymous mutations and non-hotspots (Fig. 7b). As an important target for tumor therapy [51], Ion_trans domain is critical for cell-to-cell communication and regulates multiple biological processes. However, the analysis of synonymous mutation distribution in Ion_trans domain is opposite with the previous analysis of missense mutations [46], which may be due to the different pathogenic mechanisms of synonymous and non-synonymous mutations. 7tm_1 domain is the highest proportion of hotspot mutations and consists of the group of G protein-coupled receptor, which could promote cancer metastasis [52].

## Conclusions

In this study, we not only investigated the distribution and mutational nucleotide changes of synonymous mutations across 15 cancer types, but also made the comparison of synonymous mutational signatures between TCGA and 1000G at nucleotide and amino acid levels. Meanwhile, we nominated 97 hotspot mutations in 86 genes in TCGA as potential drivers by considering their mutational rates across different mutational subtypes. And the common and diverse mutational signatures among hotspot mutations, neutral synonymous mutations of HCMGs in 1000G and non-hotspot mutations of HCMGs in TCGA were also detected. The result indicated that there were significant differences in conservation, amino acids and domain characterization between hotspots and neutral synonymous mutations. But there are some limitations in this study. Firstly, it needs more experimental work to investigate the effects of these hotspots on protein folding, RNA splicing, stability and folding, and whether they are drivers in cancers, and the relationship with cancer clinical outcome or treatment response. Secondly, the consistent patterns and specificity of hotspots in individual cancer are important and should be explored. But in this study, we just performed a pan-cancer analyzed of the hotspots. We will attack these problems in our further work. In summary, the present study would help to better understand the function of synonymous mutations in different cancer types and depicting their roles in carcinogenesis.

## Supplementary information

**Additional file 1: Figure S1.** Illustration of analysis procedure of cancer associated synonymous mutations. **Figure S2.** Correlation between percentages of synonymous mutations and codon numbers of amino acids in TCGA and 1000G. **Figure S3.** Distribution of synonymous hotspot mutations across cancer types and genes. **Table S1.** Synonymous codons of amino acids with optimal and non-optimal codons for human genome. **Table S2.** Hotspot synonymous mutations across different cancer types in TCGA dataset.

**Additional file 2: Table S3.** List of neutral synonymous mutations of HMCGs in 1000G dataset.

**Additional file 3: Table S4.** List of non-hotspot synonymous mutations of HMCGs in TCAGA dataset.

## Data Availability

The datasets supporting the conclusions of this article are included within the article and its additional files.

## Abbreviations

1000G: 1000 Genomes Project
ATts: AT transition
ATtv: AT transversion
BRCA: Breast cancer
C_GCts: CpG-island GC transition
C_GCtv: CpG-island GC transversion
CEAD: Cervical adenocarcinoma
CNST: Central nervous system tumor
ENAD: Endometrial adenocarcinoma
HLTU: Haematopoietic and lymphoid tumor
HMCGs: Hotspot-mutation-containing-genes
INAD: Large intestine adenocarcinoma
KICA: Kidney carcinoma
LICA: Liver carcinoma
LUAD: Lung adenocarcinoma
NC_GCts: Non-CpG-island GC transition
NC_GCtv: Non-CpG-island GC transversion
OVAD: Ovarian carcinoma
pos1: The first codon position
pos3: The third codon position
PRAD: Prostate adenocarcinoma
RS: Rejected substitution
SKCA: Skin cancer
SNPs: Single nucleotide polymorphisms
STAD: Stomach adenocarcinoma
TCGA: The Cancer Genome Atlas
THCA: Thyroid carcinoma
UTCA: Urinary tract
