# Supplementary material for: An analysis of mutational signatures of synonymous mutations across 15 cancer types
Source: BMC Med Genet. 2019 Dec 9;20(Suppl 2):190. doi: 10.1186/s12881-019-0926-4 (PMC6900878; doi:10.1186/s12881-019-0926-4)
Supplement: Supplementary file 1 — Additional file 1: Figure S1. Illustration of analysis procedure of cancer associated synonymous mutations. Figure S2. Correlation between percentages of synonymous mutations and codon numbers of amino acids in TCGA and 1000G. Figure S3. Distribution of synonymous hotspot mutations across cancer types and genes. Table S1. Synonymous codons of amino acids with optimal and non-optimal codons for human genome. Table S2. Hotspot synonymous mutations across different cancer types in TCGA dataset. [file 12881_2019_926_MOESM1_ESM.pdf]

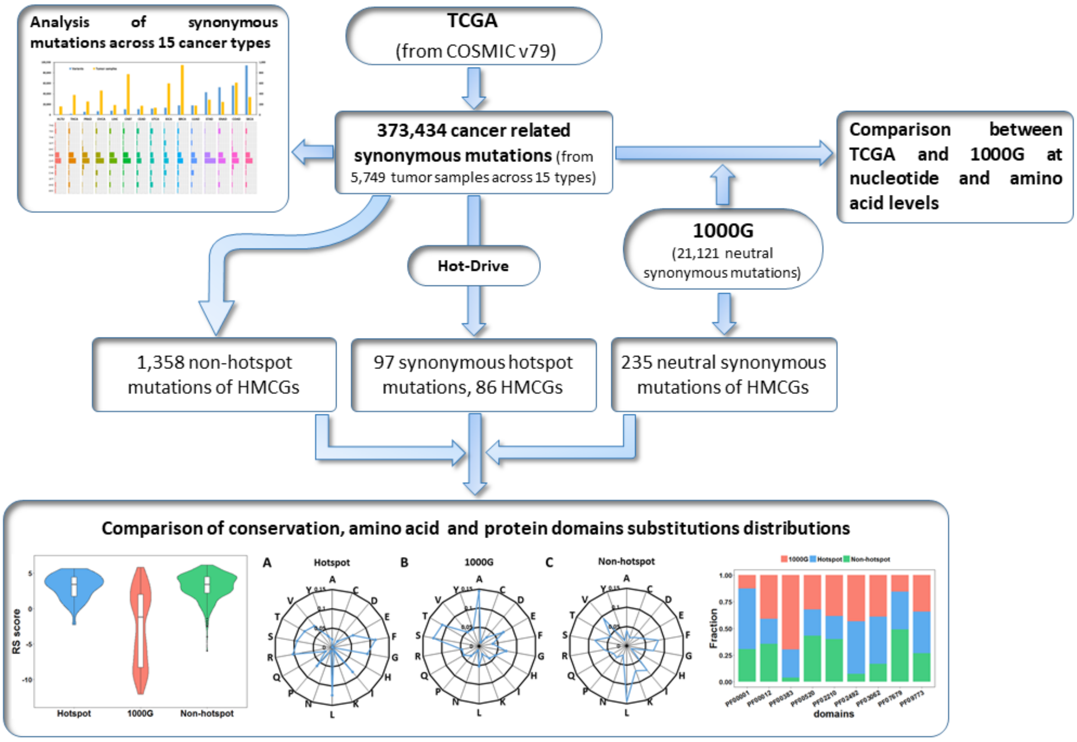

Figure S1. Illustration of analysis procedure of cancer associated synonymous mutations. Firstly, the distribution and mutational patterns of synonymous mutations from TCGA across 15 cancer types were investigated. Secondly, the comparison of synonymous mutational signatures between TCGA and 1000G at nucleotide and amino acid levels was made. Meanwhile, 97 hotspot mutations in 83 hotspot-mutation-containing-genes were nominated as potential drivers by considering the mutational rates across different mutational subtypes. And the common and diverse mutational signatures of hotspots, neutral synonymous mutations of HMCGs in 1000G and non-hotspots of HMCGs in TCGA were observed.

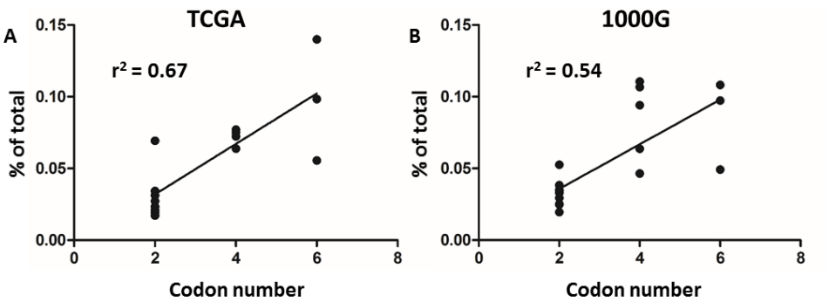

Figure S2. Correlation between percentages of synonymous mutations and codon numbers of amino acids in TCGA **(A)** and 1000G **(B)**. The x-axis represents the codon numbers of amino acids and y-axis represents the percentage of synonymous mutations.

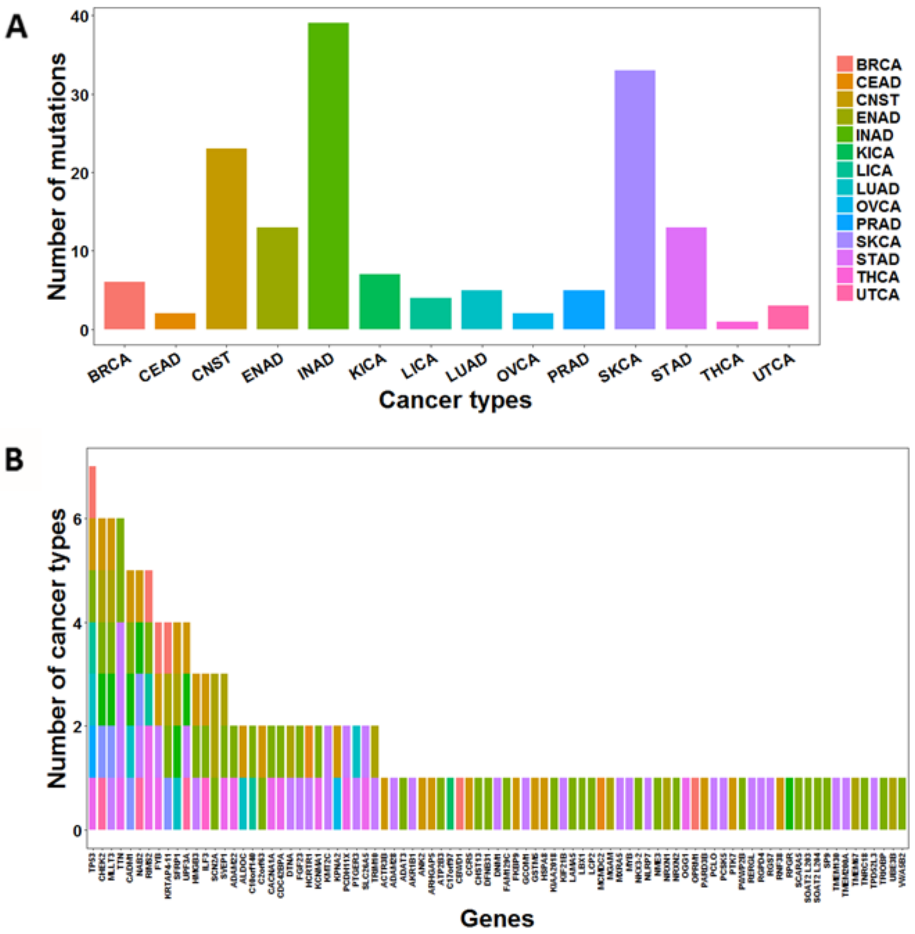

Figure S3. Distribution of synonymous hotspot mutations across cancer types **(A)** and genes **(B)**. The color bar in panel B is corresponded to the cancer types in pane A.

Table S1. Synonymous codons of amino acids with optimal and non-optimal codons for human genome (Supek F, et al. Cell. 2014;156:1324-1335).

AA	Optimal codon	Non-optimal codon	AA	Optimal codon	Non-optimal codon
**Leu (L)**	CTT	CTA	**Arg (R)**	CGT	AGA
	CTG	CTC			AGG
		TTA			CGA
		TTG			CGC
					CGG
**Ser (S)**	AGC	AGT	**Ala (A)**	GCT	GCC
	TCT	TCA			GCA
		TCC			GCG
		TCG			

**Gly (G)**	GGC	GGA<br>GGG<br>GGT	**Pro (P)**	CCT	CCA<br>CCC<br>CCG
**Thr (T)**	ACT	ACA<br>ACC<br>ACG	**Val (V)**	GTG	GTA<br>GTC<br>GTT
**Ile (I)**	ATT	ATA<br>ATC	**Cys (C)**	TGC	TGT
**Asp (D)**	GAC	GAT	**Glu (E)**	-*	GAA<br>GAG
**Phe (F)**	TTC	TTT	**His (H)**	CAC	CAG
**Lys (K)**	-	AAA<br>AAG	**Asn (N)**	AAC	AAT
**Gln (Q)**	-	CAA<br>CAG	**Tyr (Y)**	TAC	TAT

* "-" represents amino acid without optimal codon.

Table S2. Hotspot synonymous mutations across different cancer types in TCGA dataset

**Gene**	**AA position**	**Mutation count**	**Mutation subtype**	**Mutation positions (GRCh37)**	**adj.p-value**	**Cancer type**
CHEK2	S372	35	NC_CGts	22:29091841C/T	2.62E-29	CNST, ENAD, KICA, INAD, PRAD, UTCA
TP53	T125	28	NC_CGtv	17:7579312G/T	3.57E-20	INAD, LICA, LUAD, OVCA
TP53	T125	28	NC_CGtv	17:7579312G/C	3.57E-20	BRCA, STAD
FYB	A2	20	NC_CGts	5:39203057G/A	4.43E-15	BRCA, CNST, SKCA, STAD
MLLT3	S168	21	NC_CGts	9:20414340C/T	7.11E-15	CNST, ENAD, KICA, INAD, PRAD, SKCA
UPF3A	L91	19	C_CGts	13:115047559C/T	9.44E-14	CNST, KICA, SKCA, UTCA
ANK2	L1097	17	NC_CGts	4:114254274C/T	7.87E-13	CNST
MXRA5	G792	12	NC_CGts	X:3241350G/A	8.30E-09	SKCA
TTN	P68	15	NC_CGts	2:179666956C/T	2.91E-07	INAD, SKCA
FKBP9	L496	13	NC_CGts	7:33042403C/T	1.76E-06	CNST
TNRC18	R1998	16	C_CGtv	7:5372406C/A	2.01E-06	INAD
TTN	P6	14	NC_CGts	2:179669352G/A	2.10E-06	INAD, STAD
SCN2A	V1532	12	NC_CGtv	2:166243300C/A	5.85E-06	ENAD, INAD

SCN2A	V1532	12	NC_CGts	2:166243300C/T	5.85E-06	ENAD
CCR5	I67	10	NC_CGts	3:46414594C/T	1.03E-05	CNST
C10orf140	E422	12	NC_CGts	10:21805486G/A	1.05E-05	INAD, LICA
C2orf63	G39	8	NC_CGtv	2:55449431C/A	1.89E-05	CNST, INAD
KPNA2	F17	10	NC_CGts	17:66033299C/T	1.89E-05	CNST, OVCA
PWWP2B	E344	8	C_CGts	10:134219036G/A	1.89E-05	INAD
CADM1	T343	11	NC_CGtv	11:115080343C/A	2.58E-05	CNST, KICA, LUAD, PRAD, INAD
ILF3	N192	11	NC_CGts	19:10789305C/T	2.58E-05	CNST, INAD, THCA
SCARA5	L153	10	C_CGts	8:27779545G/A	3.65E-05	INAD
TRIOBP	S1295	9	NC_CGts	22:38122448C/T	5.33E-05	INAD
PTGER3	I287	12	C_CGts	1:71512400C/A	6.06E-05	LUAD
PTGER3	I287	12	C_CGtv	1:71512400C/T	6.06E-05	SKCA
TTN	R606	12	NC_CGts	2:179654825G/A	1.02E-04	SKCA
DFNB31	V39	10	C_CGts	9:117266965G/A	1.21E-04	INAD
HSPA8	G201	10	ATts	11:122930698A/G	1.94E-04	CNST
ADAM28	G411	8	NC_CGts	8:24188792G/A	2.75E-04	SKCA
GSTM5	D40	9	NC_CGts	1:110255748C/T	4.67E-04	CNST
VWA5B2	A160	7	NC_CGts	3:183951135C/T	5.05E-04	INAD
LBX1	R76	10	C_CGts	10:102988345C/T	5.62E-04	INAD
CDC42BPA	S286	10	NC_CGts	1:227335096G/A	6.47E-04	INAD, STAD
RNF38	L147	8	NC_CGts	9:36369845C/T	8.91E-04	CNST
CHST13	R71	8	C_CGtv	3:126260608G/T	1.17E-03	INAD
PCDH11X	E799	10	NC_CGts	X:91133636G/A	1.24E-03	SKCA, STAD
PCSK5	R580	9	NC_CGts	9:78784740G/A	1.37E-03	SKCA
C17orf97	T3	6	C_CGtv	17:260142G/T	2.19E-03	LIAD
KIAA2018	Q1470	7	NC_CGts	3:113376119G/A	2.19E-03	INAD
RIMS2	S201	10	ATtv(10)	8:104898096T/G	2.22E-03	BRCA, INAD, LICA, STAD
RIMS2	S201	10	ATtv(10)	8:104898096T/A	2.22E-03	STAD
KCNMA1	S504	12	NC_CGts	10:78844406G/A	2.93E-03	INAD, STAD
SOAT2	L203	7	NC_CGtv	12:53509339G/T	3.06E-03	INAD
PTK7	I763	6	NC_CGts	6:43112226C/T	3.16E-03	CNST
KRTAP4-11	P137	7	NC_CGts	17:39274157C/T	3.31E-03	BRCA, ENAD, INAD, PRAD

ATP2B3	V864	6	NC_CGtv	X:152823728G/C	4.81E-03	INAD
TMEM200A	R320	10	NC_CGts	6:130762527G/A	5.04E-03	SKCA
LAMA5	S191	6	NC_CGts	20:60927412C/T	5.53E-03	INAD
CBWD1	Y161	6	ATts	9:163985T/C	6.21E-03	BRCA
NKX3-2	R83	7	C_CGtv	4:13545792C/A	6.48E-03	INAD
ARHGAP5	K464	8	ATts	14:32561267A/G	7.65E-03	CNST
NME3	V94	8	ATtv	16:1820992A/T	7.65E-03	INAD
SOAT2	L204	7	NC_CGts	12:53509340C/T	7.88E-03	INAD
NLRP7	F333	6	NC_CGts	19:55451188C/T	7.96E-03	SKCA
GCOM1	R65	6	NC_CGts	15:57910263G/A	8.81E-03	SKCA
HMGB3	E192	9	NC_CGts	X:150156360G/A	9.27E-03	CNST, INAD, SKCA
CACNA1A	F301	10	NC_CGts	19:13470495C/T	1.01E-02	INAD, STAD
PCLO	F4094	9	NC_CGts	7:82545020C/T	1.01E-02	SKCA
DTNA	F114	10	NC_CGts	18:32374194C/T	1.06E-02	ENAD, SKCA
LCP2	V188	6	NC_CGts	5:169695446G/A	1.24E-02	INAD
RGPD4	L1578	6	NC_CGts	2:108489194G/A	1.24E-02	SKCA
ADAM22	G356	8	NC_CGts	7:87762257C/T	1.27E-02	INAD, STAD
HCRTR1	F57	6	NC_CGts	1:32084964C/T	1.27E-02	CEAD, SKCA
NAB2	P211	7	ATts	12:57485457T/C	1.27E-02	CNST, KICA, PRAD, SKCA, UTCA
SFRP1	G44	8	C_CGtv	8:41166547C/A	1.47E-02	CNST, ENAD, KICA, LUAD
SP9	G162	6	C_CGtv	2:175201299C/G	1.47E-02	INAD
KMT2C	T316	6	NC_CGts	7:151970854C/T	1.57E-02	SKCA
KMT2C	T316	6	NC_CGtv	7:151970854C/A	1.57E-02	SKCA
ADAT3	L298	5	C_CGts	19:1912986C/T	1.81E-02	INAD
SVEP1	D1461	9	NC_CGts	9:113208197C/T	1.85E-02	ENAD, INAD, STAD
ACTR3B	F131	6	ATts	7:152511691T/C	1.94E-02	CNST
TMEM67	I918	6	NC_CGtv	8:94822105C/A	1.98E-02	ENAD
KIF21B	E487	6	C_CGts	1:200969850G/A	2.03E-02	SKCA
AKR1B1	F123	5	NC_CGts	7:134134532C/T	2.06E-02	SKCA
OPRM1	Y338	8	ATts	6:154412457T/C	2.06E-02	BRCA
TPD52L3	R107	6	NC_CGts	9:6328916G/A	2.06E-02	SKCA
TTN	I1774	9	NC_CGts	2:179641269C/T	2.06E-02	SKCA

NRXN2	G177	7	C_CGts	11:64480641C/T	2.10E-02	INAD
TP53	E224	8	NC_CGts	17:7578177G/A	2.31E-02	CNST
RGS7	F347	9	NC_CGts	1:240975259C/T	2.47E-02	SKCA
RPGR	T51	4	NC_CGts	X:38182653C/T	3.10E-02	KICA
MYB	I376	6	NC_CGts	6:135517065C/T	3.19E-02	SKCA
PARD3B	G308	6	ATts	2:205986432T/C	3.19E-02	CNST
TMEM130	F265	6	NC_CGts	7:98452871C/T	3.19E-02	SKCA
MGAM	T1114	8	NC_CGtv	7:141755385C/A	3.30E-02	ENAD
DNM1	I395	6	NC_CGts	9:130985128C/T	3.43E-02	SKCA
ALDOC	L257	5	NC_CGts	17:26901115C/T	3.58E-02	CNST, LUAD
NRXN1	S211	12	C_CGts	2:51254779C/T	3.58E-02	ENAD
SLC26A5	V210	6	NC_CGts	7:103050937C/T	3.58E-02	SKCA, STAD
FAM129C	Q502	4	NC_CGts	19:17654209G/A	3.65E-02	INAD
MCMDC2	Y319	4	NC_CGts	8:67796113C/T	3.76E-02	CEAD
RERGL	R202	6	NC_CGts	12:18234137G/A	3.79E-02	SKCA
UBE3B	L83	7	NC_CGts	12:109921751C/T	3.87E-02	ENAD
FGF23	F108	5	C_CGts	12:4479941C/T	4.03E-02	INAD, SKCA
OGG1	L201	5	NC_CGtv	3:9796425G/C	4.83E-02	STAD
TRIM10	P71	6	NC_CGts	6:30128423C/A	4.83E-02	ENAD
TRIM10	P71	6	NC_CGtv	6:30128423C/T	4.83E-02	SKCA
